# Structural and Viability Assessment of Bovine Corneas Preserved at an Accessible Low-Temperature (−20 °C) for Eye Irritation Models

**DOI:** 10.1021/acsomega.5c04314

**Published:** 2025-10-03

**Authors:** Geovana Onorato, Janildo Ludolf Reis Junior, Humberto Mello Brandão, Michele Munk

**Affiliations:** a Laboratory of Nanobiotechnology and Nanotoxicology, Department of Biology, Federal University of Juiz de Fora, Juiz de Fora, Minas Gerais 36036-900, Brazil; b Department of Veterinary Medicine, Federal University of Juiz de Fora, Juiz de Fora, Minas Gerais 36036-900, Brazil; c Laboratory of Applied Nanotechnology for Animal Production and Health, Brazilian Agricultural Research Corporation, Juiz de Fora, Minas Gerais 36038-330, Brazil

## Abstract

*In vitro* ocular irritation testing requires the use of freshly excised bovine corneas, which limits broader adoption in laboratories with restricted access to slaughterhouse material. This study investigated a practical and accessible low-temperature preservation strategy focused on maintaining structural and cellular integrity. Bovine corneas were stored at −20 or −80 °C using two commonly available cryoprotective agents: dimethyl sulfoxide (DMSO) and ethylene glycol (EG). Preservation outcomes were assessed through corneal transparency, histological architecture, and endothelial viability. Among the tested conditions, storage at −20 °C with 10% DMSO provided the most consistent preservation of structural morphology and transparency. This protocol provides a cost-effective foundation for extending the usability of excised corneas and supports future efforts to expand the accessibility of *in vitro* eye irritation models in laboratories with limited infrastructure.

## Graphical abstract

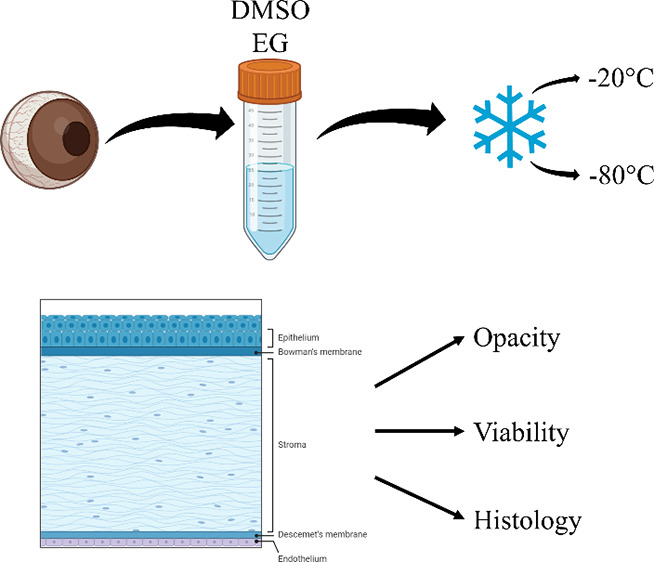

## Introduction

Toxicological assessment of new chemical compounds and pharmaceutical formulations is a fundamental step in ensuring their safety for both occupational and consumer exposure, supporting regulatory compliance and public health protection. Traditionally, *in vivo* assays have served as the gold standard for predicting adverse effects prior to human exposure. However, these models present significant ethical and scientific limitations. *In vivo* tests often cause animal suffering and are frequently inconsistent with clinical outcomes, exhibiting low reproducibility and limited ability to model human pathophysiology accurately.^1,2^ Moreover, the translational relevance of animal models is increasingly questioned due to interspecies biological and physiological differences.^3^ These challenges have accelerated the development of *in vitro* assays, which offer advantages in terms of predictive accuracy, reproducibility, and human relevance.

Within this context, the bovine corneal opacity and permeability (BCOP) test was developed as an alternative to the Draize eye irritation test in rabbits.^4,5^ The BCOP assay, which evaluates the ocular irritation potential of chemicals, including both pure substances and mixtures, by quantifying corneal opacity and permeability, has been validated and is formally recognized by the Organisation for Economic Cooperation and Development (OECD).^6^ It is frequently used in combination with other *in vitro* tests to determine chemical hazard classification.^7^ A major advantage of the BCOP test is the accessibility of bovine corneas, which are byproducts of slaughterhouses where animals deemed fit for human consumption also provide suitable tissues for toxicological assessments.^7,8^ However, the reliance on fresh corneas presents a critical logistical limitation, as tissues must be used immediately after extraction to preserve biological integrity. This highlights the urgent need for a standardized long-term preservation strategy to secure a stable supply of corneas for BCOP testing.

Preservation methods for human corneas have been extensively optimized, particularly in transplantation contexts.^9^ Cryopreservation, which employs ultralow temperatures to arrest biological activity and prevent tissue degradation, has emerged as a leading approach.^10^ Typically, this requires storage in liquid nitrogen (−196 °C) or at −80 °C in ultralow freezers.^11^ However, the cost and technical requirements of these systems may limit their adoption in routine *in vitro* toxicology. In this scenario, evaluating the feasibility of preserving bovine corneas at −20 °C, a temperature achievable in standard laboratory freezers, is of particular relevance.

Low-temperature preservation efficacy depends on cryoprotective agents (CPAs), which preserve cellular integrity by preventing ice crystal formation, stabilizing osmotic balance, and reducing mechanical stress during freezing and thawing. Among the most widely used CPAs, dimethyl sulfoxide (DMSO, C_2_H_6_OS) and ethylene glycol (EG, C_2_H_6_O_2_) exhibit distinct physicochemical properties that influence their interactions with biological tissues. DMSO, due to its amphiphilic nature, diffuses rapidly across cell membranes, promoting efficient intracellular equilibration and minimizing mechanical damage from ice formation.^12,13^ EG, a smaller polyol, acts by disrupting hydrogen bonding between water molecules, thus lowering the freezing point. However, its higher viscosity and hydrophilicity influence permeability and water dynamics differently from DMSO.^14^

Despite extensive literature on cryopreservation protocols for human corneas, direct application to bovine tissues is limited by significant anatomical and physiological differences.^15^ The bovine corneal stroma is considerably thicker than that of humans, measuring approximately 844 μm at the center and exceeding 1000 μm in the peripheral regions, compared to the human cornea, which measures 500–600 μm.^16^ This thickness is linked to higher collagen content and extracellular matrix density, particularly in peripheral regions.^16,17^ The collagen fibril architecture also differs, with bovine corneas exhibiting more uniform fibril diameter across regions.^18^ Additionally, bovine endothelial cells have lower density and more irregular morphology, factors that may influence hydration and optical transparency.^19^ These structural differences are essential considerations in comparative ocular research and applications, particularly for *in vitro* toxicological assessments and preclinical models of corneal physiology. These factors necessitate the development of tailored low-temperature preservation methods to ensure tissue integrity and functional viability for accurate and reliable BCOP testing. Thus, the objective of this study is to evaluate preservation methods capable of maintaining bovine corneal integrity and viability for consistent application in BCOP assays.

## Materials and Methods

### Materials

Alizarin Red, bovine serum (BS), bovine serum albumin (BSA), dimethyl sulfoxide (DMSO), Dulbecco’s modified Eagle’s medium (DMEM), eosin, ethylene glycol (EG), formalin, hematoxylin, iodopolyvidone (I-PVP), phosphate-buffered saline (PBS), sodium chloride (NaCl), sodium thiosulfate, and Trypan Blue were obtained from Sigma-Aldrich (St. Louis, MO, USA).

### Collection of Bovine Corneas

A total of 180 bovine corneas were collected post-mortem from a licensed slaughterhouse in Juiz de Fora, Brazil, following standardized procedures to ensure tissue integrity. No live animals were used in this study, and all tissues were handled in accordance with ethical guidelines for the use of animal byproducts in research. The enucleated eyes were immediately immersed in chilled PBS 1× and transported to the laboratory in an insulated thermos maintained at 10 °C to minimize tissue degradation. Upon arrival, the eyes were subjected to macroscopic evaluation based on pre-established inclusion criteria, with only corneas free of mechanical trauma, incisions, excessive opacity, or neovascularization being selected for further processing. The excision protocol was adapted from methodologies established for human corneal retrieval to optimize handling and preservation of bovine tissues.^20^ Selected eyes were transferred to a laminar flow cabinet for a sterilization procedure aimed at minimizing microbial contamination. The decontamination process consisted of sequential immersion in 0.5% I-PVP solution (v/v) for 2 min, followed by immersion in 0.1% sodium thiosulfate (w/v) for 1 min to neutralize residual iodine, and a final rinse in sterile PBS 1× before corneal excision. Under aseptic conditions, corneas were excised using a sterile razor blade and microscissors. An initial incision was made approximately 2 mm beyond the limbus, preserving a protective scleral rim to facilitate handling and maintain tissue integrity. Corneas were then gently dissected and transferred to the appropriate experimental conditions for subsequent low-temperature preservation and evaluation.

### Swelling Test

The swelling test was performed to assess corneal mass variation following exposure to CPAs and to evaluate endothelial tolerance and integrity in response to these substances.^21^ Following excision, the corneas (*n* = 54) were carefully separated from the iris, and residual aqueous humor was removed with sterile filter paper. The initial weight of each cornea was recorded using an analytical balance (SHIMADZU AUX 220). Subsequently, corneas were exposed to different CPA treatments in DMEM without phenol red.

For the treatment groups, corneas were exposed to two CPAs: DMSO (at 7.5, 10, and 15%) and ethylene glycol (EG; at 4, 8, and 12%). Each concentration was prepared in a basal medium (DMEM) supplemented with either 5% BSA, 5% BS, or no adjuvant. A comprehensive set of control groups was also included, consisting of pure DMEM (vehicle control) and DMEM supplemented with either 5% BSA or 5% BS to assess their independent effects. Additionally, a 10% BSA control was incorporated to evaluate the corneal response to a higher adjuvant concentration.

Corneal weight measurements were conducted at 0, 2, 4, 6, 8, and 24 h postexposure to CPAs. To normalize the data and account for intersample variability, corneal weights were expressed as a percentage relative to the initial weight of each sample, allowing for the evaluation of weight gain or loss over time. CPA concentrations that resulted in a more uniform swelling response were selected for further analysis in subsequent tests. The selection of DMSO and EG as cryoprotective agents in this study was guided by their widespread use in tissue cryopreservation and their contrasting physicochemical properties, particularly regarding membrane permeability and osmotic behavior.

### Freezing Procedure

The selected cryoprotectant concentrations for the freezing step were 10% DMSO and 8% EG, both supplemented with 10% BS in DMEM medium. To facilitate osmotic equilibration and minimize cryoinjury, corneas (*n* = 108) were gradually exposed to increasing CPA concentrations: DMSO was applied in 3, 7, and 10% solutions, while EG was introduced at 3, 6, and 8%. Each equilibration step was performed for 5 min. After equilibration, each cornea was transferred to a 50 mL conical centrifuge tube containing 30 mL of the final cryoprotectant solution, ensuring that the tissue was completely immersed. The samples were then stored at either −20 or −80 °C for a fixed period of 30 days according to the assigned experimental group. After storage, corneas were thawed and processed for analysis.

### Thawing Procedure

Thawing of corneas previously frozen at low temperature was performed in a 42 °C water bath until the scleral tissue was completely thawed. Corneas were then immediately transferred to a defrosting solution composed of DMEM supplemented with 5% of the respective CPA and maintained at 37 °C until complete thawing and CPA dissociation. Subsequently, corneas were transferred to fresh DMEM at room temperature to remove residual CPAs before further analyses.^22^

### Macroscopic Analysis

Following thawing, bovine corneas (*n* = 63) underwent macroscopic analysis to assess tissue transparency and opacity, following methodologies previously described in the literature.^23^ Corneal opacification was evaluated by a single trained observer to ensure consistency in assessments. The degree of opacity was classified based on the Ashworth et al.^24^ scale, with modifications, into three distinct categories: (i) low, (ii) mild, and (iii) high opacity.

### Endothelial Viability Test

The integrity of endothelial cell membranes and viability were assessed in fresh corneas (*n* = 9) and in samples stored at −20 °C (*n* = 27) and −80 °C (*n* = 27). A dual-staining protocol was employed using 0.25% (w/v) Trypan Blue to evaluate membrane integrity and 0.2% (w/v) Alizarin Red S to identify endothelial cells with calcium deposition, which may reflect late-stage cellular injury or necrosis. Quantification was performed using an inverted light microscope (Zeiss Primo Vert, Oberkochen, Germany) following the protocol described by Camposampiero et al.^25^ After macroscopic analysis, the corneal endothelium was sequentially exposed to Trypan Blue for 30 s, followed by washing in 0.9% NaCl solution. The tissue was then exposed to Alizarin Red for 20 s, rinsed again in 0.9% NaCl, and positioned in a Petri dish with the endothelial layer facing downward to facilitate imaging. Micrographs were captured at 400× magnification under a light microscope, and endothelial viability was assessed by counting viable cells in three distinct fields, in different regions of the tissue, without overlapping. Quantification was performed using ImageJ software, allowing for standardized and reproducible cell viability analysis.

### Endothelial Cell Morphometry and Morphology

The mean endothelial cell area (μm^2^) was determined from corneas stained with Trypan Blue and Alizarin Red, following the methodology described by Ewete et al.^26^ A total of *n* = 60 cells per treatment group were analyzed using the Carl Zeiss Microscopy ZEN 2.3 (Blue Edition) software to obtain precise morphometric measurements. In addition to quantitative assessment, the cellular morphology was evaluated qualitatively, considering structural integrity, cellular borders, and the presence of polymegathism or pleomorphism as indicators of endothelial preservation across different low-temperature preservation conditions.

### Histological Analysis

Histological sections were prepared to assess the integrity of corneal cells, corneal membranes, and stromal structure following low-temperature preservation. After thawing, corneas were fixed in 10% buffered formalin, embedded in paraffin, and sectioned longitudinally at a thickness of 4–5 μm to allow visualization of the corneal cellular layers and overall tissue histoarchitecture. The nuclei and cytoplasm of the histological sections were stained using hematoxylin–eosin (HE) for structural analysis.^27^ Corneal integrity was evaluated based on a semiqualitative classification system adapted from Costa et al.^28^ The classification categories included (i) absent, (ii) minimal, (iii) discrete, (iv) discrete to moderate, (v) moderate, (vi) moderate to accentuated, and (vii) accentuated. Frequency data were expressed as percentages, with a sample size of *n* = 9 per treatment group.

### Statistical Analysis

Data from the swelling test and endothelial viability assay were analyzed using linear models implemented in the Stats package with the LM.^29^ Prior to model selection, collinearity between explanatory variables was assessed to eliminate redundant predictors and ensure model robustness. Model selection was performed based on the Akaike Information Criterion corrected for small samples (AICc),^30^ with ΔAICc < 2 used to identify the most parsimonious models. Among the selected models, those with the lowest AICc values were preferred. For cell viability data, the Kruskal–Wallis test was applied to assess differences among treatment groups, followed by pairwise comparisons using the Wilcoxon test where applicable. The frequency distribution of categorical corneal opacity scores (i.e., low, mild, and high) across treatment groups was analyzed using a chi-squared (χ^2^) test of independence. All statistical analyses were conducted using R software,^29^ and *p*-values <0.05 were considered statistically significant.

## Results and Discussion

### Swelling Test

Ensuring a reliable supply of preserved bovine corneas is essential for expanding the applicability of the BCOP assay, a validated *in vitro* method recognized by the OECD for assessing the ocular safety of chemical and pharmaceutical products. This study aimed to develop a practical and cost-effective preservation strategy capable of maintaining corneal integrity and viability under low-temperature freezing. By comparing DMSO and EG at −20 and −80 °C, we sought to overcome a key logistical barrier to the routine use of BCOP in laboratories with limited resources. As a first step, corneal swelling behavior was assessed as an indicator of osmotic balance and tissue integrity following exposure to various CPAs and adjuvants. Corneas were exposed to varying concentrations of DMSO and EG, either supplemented with BS or BSA or used alone. Weight variation over 24 h was analyzed using linear models that included treatment (Trat), treatment concentration (Conc_t), adjuvants (Adj), and time (T) as predictors, with percentage weight change (Por) as the response variable. Model selection was based on AICc (see Supporting Information, Table S1), and the best-fitting model, which incorporated all predictors and their interactions, explained approximately 91% of the observed variability (*R* ^2^ = 0.91; Figure S1).

As shown in Figure 1, the swelling test revealed a consistent initial weight loss within the first 2 h across all CPA-treated groups. This phenomenon is likely attributable to CPA permeability and the associated osmotic dehydration of the corneal tissue, as previously described.^31^ DMSO-treated corneas exhibited greater weight loss than those treated with EG, consistent with DMSO’s higher permeability in corneal tissues^32^ and its faster intracellular penetration.^33^ This behavior parallels observations in human oocytes^34^ and Jurkat cells.^35^

**Figure 1:**
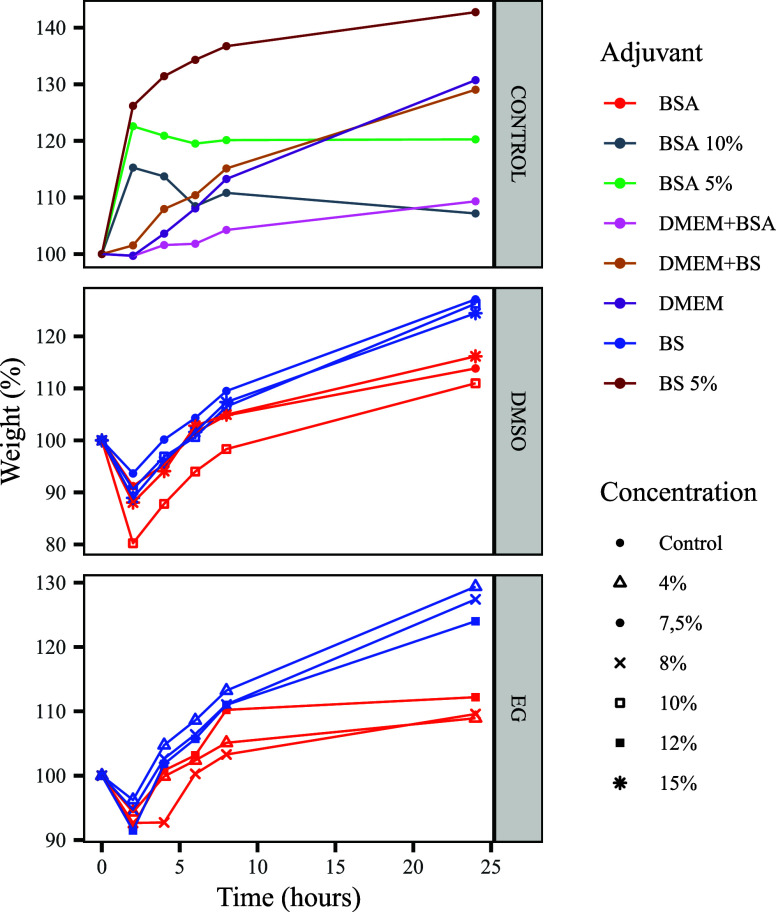
Weight variation (%) of bovine corneas over 24 h after exposure to different cryoprotective agents (CPAs). The figure is divided into three panels: control treatments (top), DMSO-treated corneas (middle), and EG-treated corneas (bottom). Adjuvants (BSA, BS, DMEM + BSA, and DMEM + BS) are indicated by different colors, and CPA concentrations (4, 7.5, 8, 10, 12, and 15%) are shown with distinct symbols. CPA-treated corneas exhibited an initial weight loss within the first 2 h, followed by progressive recovery dependent on the CPA type, adjuvant, and concentration.

After this initial phase, the weight progressively increased and stabilized by 24 h, indicating osmotic recovery through CPA redistribution and water reabsorption. EG’s rapid equilibration^36^ may support this recovery process but can also provoke osmotic stress if not properly managed.^33^ CPA-treated corneas significantly differed from controls (*p* < 0.05), and adjuvants BS and BSA also showed a significant impact (*p* < 0.05), jointly accounting for ∼32% of swelling variation (*R* ^2^ = 0.32). BSA, in particular, stabilized membranes and mitigated osmotic stress,^37^ while its antioxidative properties reduced lipid peroxidation and oxidative injury.^38^ These protective effects are reflected in the reduced weight variation of BSA-treated corneas (*p* = 0.43), as further supported by Nang et al.^39^ and Kolyada et al.^40^

Time emerged as a critical factor, explaining ∼39% of the total variation (*R* ^2^ = 0.39). The post-2 h weight increase may indicate CPA-induced cytotoxicity or late-phase osmotic stress, particularly relevant in the context of EG’s hydrophilic nature.^41^

Based on these swelling profile results, the 10% DMSO and 8% EG concentrations were selected for subsequent cryopreservation experiments, as they demonstrated the most stable and predictable osmotic response, suggesting a lower risk of stress-induced tissue damage during freezing and thawing.

### Macroscopic Analysis and Endothelial Viability

After thawing, corneal transparency was assessed by categorizing samples into predefined opacity levels: low, mild, or high. Representative images of each category are provided in Figures S2 and S3. All cryopreserved corneas exhibited some degree of opacity, with DMSO-treated samples retaining greater transparency than those treated with EG. Corneas stored at −20 °C also showed better preservation than those stored at −80 °C, likely due to slower freezing rates reducing ice crystal formation. Increased opacity corresponded with reduced endothelial viability, particularly in EG-treated samples at −80 °C, where structural compromise was more evident. These findings are consistent with previous reports linking cryoinjury to intracellular and extracellular ice formation, as well as osmotic imbalance.^23,42^

The distribution of corneal opacity scores was significantly influenced by both the type of CPA and the storage temperature (*p* = 0.001; Figure 2). Corneas cryopreserved with 10% DMSO predominantly exhibited low to mild opacity, with ∼55% of samples classified as having low opacity at both freezing temperatures (−20 and −80 °C). In contrast, EG-treated corneas (8%) displayed a markedly higher degree of opacity, with over 60% of samples showing severe opacification. The increased opacity in EG-treated corneas aligns with previous research indicating that EG’s high membrane permeability may exacerbate osmotic stress, leading to localized dehydration and intracellular ice formation.^36^ Additionally, corneas stored at −80 °C exhibited greater opacification than those at −20 °C, likely due to prolonged exposure to critical temperature ranges during freezing and thawing.^43,44^

**Figure 2:**
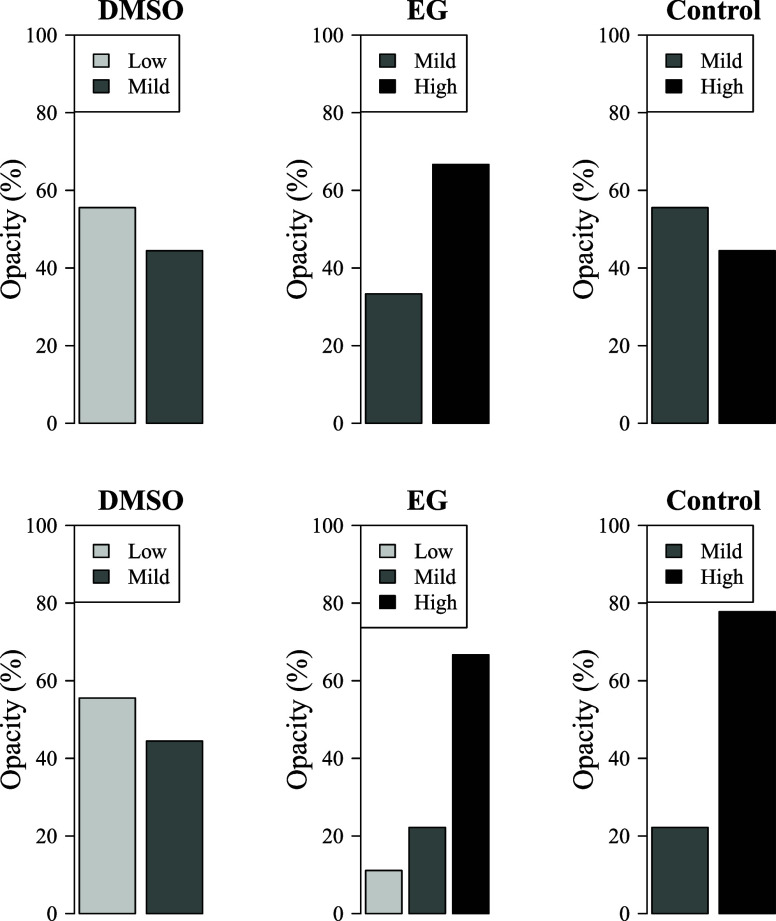
Distribution of corneal opacity scores across different preservation protocols. The 100% stacked bar chart shows the percentage of corneas (*n* = 9 per group) classified into low, mild, or high opacity categories. The top row presents results for samples stored at −20 °C, and the bottom row shows those stored at −80 °C. A chi-square test revealed a significant association between the treatment protocol and opacity score distribution (χ^2^(10) = 28.80, *p* = 0.0013).

Endothelial viability was assessed using the Trypan Blue and Alizarin Red staining, complementing the macroscopic observations. Among the experimental conditions, corneas treated with DMSO and stored at −20 °C exhibited the best preservation of membrane integrity within the frozen groups, as indicated by limited dye uptake and relatively well-maintained structural appearance (Figure S4). Although some staining was observed, the overall pattern suggests moderate preservation of endothelial viability. In contrast, samples stored at −80 °C showed markedly greater Trypan Blue penetration, reflecting increased cellular damage and loss of membrane integrity across treatments. These findings align with previous studies demonstrating that slower freezing rates, such as those associated with −20 °C, help reduce cellular injury by minimizing abrupt phase transitions during critical thermal intervals.^45,46^

All low-temperature frozen groups exhibited a statistically significant reduction in endothelial cell viability compared to fresh controls (*p* < 0.05, Figure 3). Among them, the DMSO −20 °C protocol performed best, achieving the highest mean viability. However, this difference was not statistically significant when compared to the no-CPA control group, as both showed comparable viability levels.

**Figure 3:**
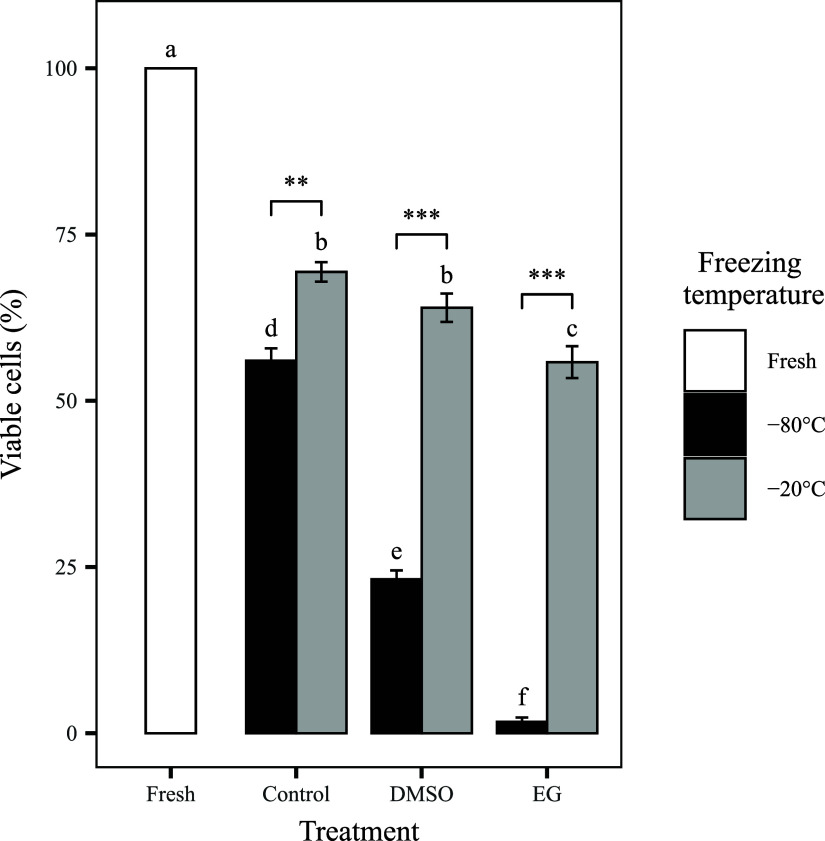
Viability of endothelial cells after low-temperature freezing at −20 and −80 °C. Fresh corneas exhibited the highest viability. Among cryopreserved groups, DMSO-treated corneas stored at −20 °C retained the greatest proportion of viable cells. Bars represent means ± SEM (*n* = 9 per group). Different letters indicate statistically significant differences (*p* < 0.05). Additional significance is denoted by **p < 0.01 and ***p < 0.001.

In contrast, all other preservation protocols resulted in markedly reduced viability. Particularly, corneas treated with EG and stored at −80 °C exhibited the most pronounced loss, with fewer than 10% of endothelial cells remaining viable, consistent with previous findings on EG’s cytotoxic effects.^47^ A linear regression model confirmed that the interaction between the CPA type and storage temperature was the main factor driving these outcomes, accounting for approximately 93% of the observed variability (*R* ^2^ = 0.9264).

These observed differences in endothelial viability and morphological integrity can be attributed to the distinct physicochemical properties and cellular interactions of DMSO and EG. Characterized by its low molecular weight, EG rapidly permeates cellular membranes, potentially causing acute intracellular osmotic disturbances and increased cytotoxicity.^31^ This high permeability facilitates rapid water displacement, which, when combined with suboptimal freezing conditions, may lead to excessive osmotic stress and structural damage. In contrast, DMSO, while similarly permeable, stabilizes cellular membranes by mitigating intracellular ice nucleation and reducing oxidative stress through its antioxidative properties.^32^ These biochemical properties likely account for the superior performance of DMSO in preserving endothelial integrity, particularly at −20 °C, where cryoinjury was less pronounced.

Endothelial damage was further assessed through dual staining with Trypan Blue and Alizarin Red. The control groups (Figure 4B,C), representing cryopreserved corneas without cryoprotectants, exhibited marked disruption, whereas corneas preserved with DMSO at −20 °C (Figure 4D) showed the best relative preservation among the cryopreserved groups. Although not free from injury, these samples maintained more defined cell borders, partial retention of the hexagonal pattern, and less diffuse dye uptake compared with the cryopreserved controls and the EG-treated groups (Figure 4F,G). By contrast, EG-treated corneas, particularly those stored at −80 °C (Figure 4G), displayed widespread staining, irregular cell borders, and nearly complete loss of hexagonal organization. The reproducibility of these patterns across multiple micrographs, integrated with the metabolic viability assay (Figure 3), quantitative cell area analysis (Figure 5), and histological evaluation (Figure 6 and Tables S2–S5), consistently supports that the DMSO −20 °C protocol achieved superior preservation of endothelial structure.

**Figure 4:**
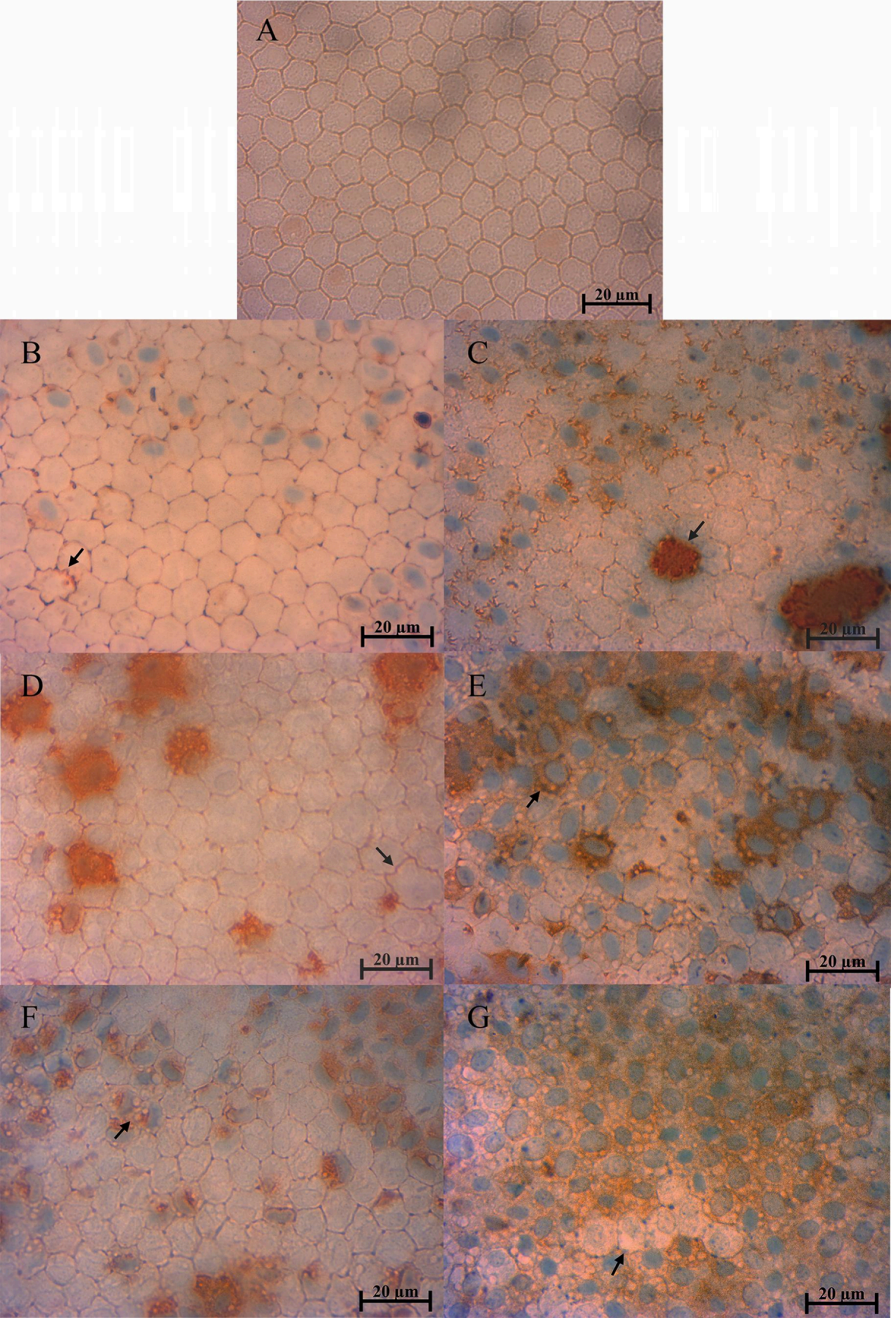
Representative micrographs of the corneal endothelium from fresh samples (A) and cryopreserved corneas stored at −20 °C (B, D, and F) or −80 °C (C, E, and G), stained with Trypan Blue and Alizarin Red. (A) Fresh corneas show a uniform hexagonal endothelial pattern with minimal staining. (B, C) Cryopreserved controls without cryoprotectants exhibit localized membrane damage. (D, E) Corneas treated with DMSO, particularly at −20 °C (D), demonstrate a relatively preserved endothelial architecture, with more defined borders and a less diffuse staining pattern compared with other cryopreserved conditions. (F, G) Samples treated with ethylene glycol (EG), especially at −80 °C (G), display widespread staining, severe disruption of the hexagonal organization, and irregular cell borders. The images shown are representative of multiple fields analyzed, all of which displayed consistent patterns. Arrows indicate regions of membrane damage. Scale bars: 20 μm.

**Figure 5:**
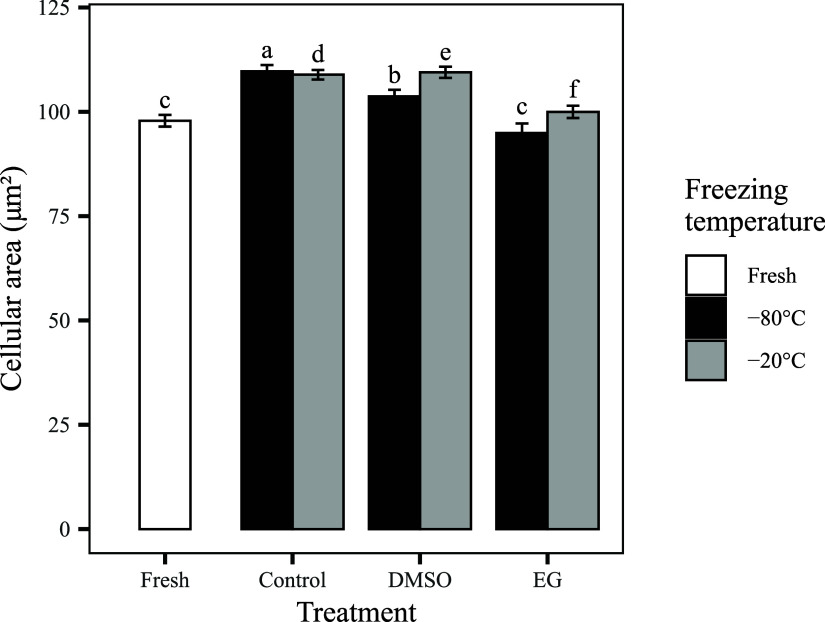
Mean cellular area (μm^2^) of the corneal endothelium in fresh corneas and after cryopreservation under different treatment conditions (control, DMSO, and EG) at −20 °C (gray bars) or −80 °C (black bars). Fresh corneas (white bar) exhibited the smallest cell area. All cryopreserved groups showed significant cellular enlargement. Bars represent means ± SEM. Statistically significant differences between groups are indicated by different letters (*p* < 0.05).

**Figure 6:**
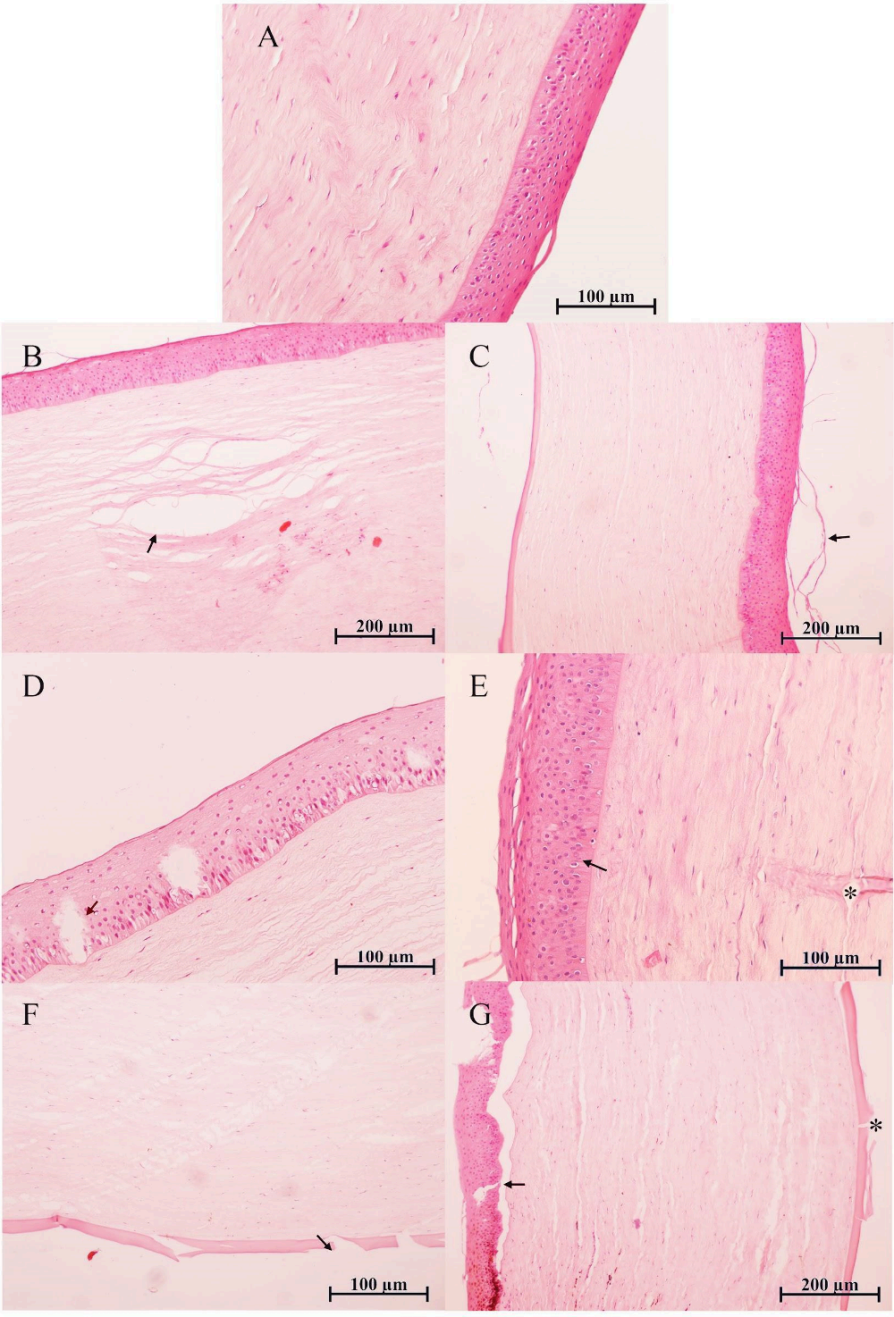
Representative histological sections of fresh (A) and DMSO-treated corneas after low-temperature freezing at −20 °C (B, D, and F) and −80 °C (C, E, and G), stained with hematoxylin and eosin (HE). (A) Fresh corneas show normal histological architecture. (B, D, and F) Corneas treated with DMSO and stored at −20 °C exhibit mild to moderate alterations, such as moderate stromal clefts (B), mild epithelial vacuolization with slight pseudokeratinization (D), and limited Descemet’s membrane detachment (F). (C, E, and G) At – 80 °C, DMSO-treated corneas showed more severe histological damage, such as epithelial detachment (C), persistent stromal clefts (E), and pronounced Descemet’s membrane detachment (G). Arrows and asterisks indicate affected regions. Scale bars: 100 or 200 μm, as shown in each panel.

In contrast, corneas exposed to 8% EG and stored at −80 °C exhibited the most severe tissue damage, as supported by an integrated analysis of multiple indicators. The micrograph for this group (Figure 4G) shows profound endothelial disruption, including loss of hexagonal organization, irregular cell borders, and intense dye uptake, consistent with advanced necrosis. Interestingly, the mean cell area for this group (Figure 5) was not larger than that of DMSO −20 °C, and in fact approximated fresh corneas. We interpret this not as evidence of preservation, but as a marker of end-stage injury, where initial edema is followed by cellular fragmentation and detachment, leading to underestimation of enlargement in the remaining population. This interpretation is supported by the metabolic viability assay (Figure 3), which showed the lowest viability in the EG −80 °C group, and is further corroborated by the histological evaluation (Tables S2–S5 and Figure 6), presented in the following section. In contrast, DMSO −20 °C corneas showed only mild to moderate alterations. Together, these findings confirm that EG −80 °C induced the most severe overall damage. These converging observations align with previous reports identifying osmotic imbalance and cryoprotectant-induced cytotoxicity as key drivers of cryoinjury at low temperatures, with the cytotoxic effects of EG being potentially intensified by its rapid membrane permeability, which amplifies osmotic stress during the freeze–thaw process.^31,47,49^

### Histology

Histological analysis of the control group stored at −20 °C revealed a gradient of cryoinjury across the corneal layers. In the epithelium, the superficial layers exhibited minimal detachment (Figure S5B), slight pseudokeratinization, and noticeable keratinocyte vacuolation (Table S2 and Figure S5F). The underlying basal epithelial layer showed distinct cellular retraction and cytoplasmic deformity (Table S3). At greater depth, the stromal region exhibited focal unstained cleft formation (Table S4 and Figure S5D), and finally, the endothelium displayed partial detachment of Descemet’s membrane (Table S5). These structural changes are indicative of extracellular ice formation and osmotic stress due to the absence of intracellular CPAs, in agreement with previous reports.^43,44^

In contrast, the control group stored at −80 °C presented a different profile of histological alterations. While epithelial detachment remained minimal, significant damage was noted in the endothelium, including distinct cell detachment (Figure S5C). In the stroma, the formation of unstained clefts persisted (Table S4 and Figure S5E), and the detachment of Descemet’s membrane was comparable to that observed at −20 °C (Table S5 and Figure S5G). These findings suggest that while lower temperatures may exacerbate specific histological alterations, they may also mitigate others, highlighting the complex interplay between freezing rates and tissue response.

Histological damage in cryopreserved corneas, including epithelial detachment, stromal clefts, and Descemet’s membrane disruptions, directly affects corneal transparency and endothelial function, both of which are critical parameters for the accuracy of the BCOP assay. These structural modifications could alter corneal permeability and optical clarity, potentially leading to false-positive or false-negative outcomes in irritancy testing. Therefore, minimizing histological damage through optimized CPA selection and freezing protocols is essential to ensure the reproducibility and reliability of BCOP assays.

Among CPA-treated samples, DMSO-treated corneas stored at −20 °C exhibited minimal epithelial alterations, such as slight pseudokeratinization and keratinocyte vacuolation (Table S2 and Figure 6D), while the basal layer displayed moderate cytoplasmic deformation (Table S3). The stroma presented with moderately pronounced clefts (Table S4 and Figure 6B), and the endothelium showed only slight Descemet’s membrane detachment (Table S5 and Figure 6F). These findings indicate a relatively well-preserved architecture and corroborate previous reports on DMSO’s efficacy in reducing cryoinjury.^48,49^

In contrast, DMSO-treated corneas stored at −80 °C displayed more pronounced damage. This included significant epithelial detachment (Figure 6C) and increased cytoplasmic deformity in the basal keratinocytes (Table S3). While stromal cleft formation remained moderate (Table S4 and Figure 6E), the detachment of Descemet’s membrane in the endothelium was notably more severe when compared to the −20 °C group (Table S5 and Figure 6G). These findings align with prior studies suggesting that faster freezing can exacerbate CPA-induced cytotoxicity.^43,46^

The EG-treated corneas generally exhibited more extensive histological damage compared to the DMSO groups. At −20 °C, this included moderate-to-marked epithelial detachment (Table S2 and Figure S6B) and significant vacuolation of both superficial and basal keratinocytes (Table S3 and Figure S6F). Furthermore, the stroma presented with moderate-to-marked cleft formation (Table S4 and Figure S6D), and the endothelium displayed moderate to accentuated Descemet’s membrane detachment (Table S5). These findings indicate that EG’s rapid penetration can lead to significant osmotic stress even at this higher freezing temperature.^33,36^

At −80 °C, the damage to EG-treated corneas was even more pronounced. Severe epithelial disorganization and basal keratinocyte vacuolation were observed (Table S3 and Figure S6C). The most critical damage occurred in the deeper layers, with extensive Descemet’s membrane detachment (>50%) and severe endothelial disorganization evident in the micrographs (Table S5 and Figure S6E,G). Previous studies have shown that rapid freezing can lead to increased osmotic and mechanical stress, resulting in structural disruptions such as Descemet’s membrane detachment. For instance, Guo et al.^49^ reported that the extent of Descemet’s membrane detachment is closely related to surgical factors and the skillfulness of the procedure, suggesting that mechanical stress plays a significant role in such detachments.

Overall, slow freezing at −20 °C resulted in reduced structural damage across all corneal layers, supporting the hypothesis that controlled freezing rates allow for better CPA equilibration and reduced intracellular ice formation.^31,43^ In contrast, rapid freezing at −80 °C induced significant osmotic and mechanical stress, leading to greater structural disruption. These findings emphasize the need for tailored low-temperature preservation protocols that optimize CPA penetration and freezing dynamics to minimize osmotic stress and tissue damage. Further refinement of CPA concentrations and equilibration procedures is crucial to enhance corneal low-temperature preservation outcomes, particularly for alternative toxicity testing applications.

An important observation in the findings is the divergence between the metabolic viability assay (Figure 3) and the complementary assays assessing structural and membrane integrity. Although the metabolic assay did not show a statistically significant advantage of the DMSO at −20 °C protocol over the no-CPA control, this result may reflect a balance between cryoprotective effects and DMSO-induced cytotoxicity under the specific assay conditions. Thus, this observation must be interpreted in the context of the broader data set. The DMSO −20 °C protocol consistently preserved key end points of the BCOP assay, including corneal transparency, histological architecture, and endothelial integrity, with minimal evidence of necrosis. This enhanced preservation is likely related to the physicochemical properties of DMSO, such as its high membrane permeability and antioxidative capacity, which help prevent intracellular ice formation and mitigate oxidative damage.^32,48^ Given these attributes, this preservation method may have broader applicability, including in ophthalmic drug testing, regenerative medicine, and *ex vivo* corneal models for transplantation studies.

This study establishes the efficacy of a 30-day corneal preservation protocol at −20 °C using DMSO, offering a practical solution to the limited availability of fresh bovine corneas. Importantly, the ability to store corneas at −20 °C, rather than −80 °C, represents a major logistical advantage by significantly reducing infrastructure requirements. This makes BCOP assays more accessible to laboratories with limited cryogenic storage capacity and supports the broader adoption of *in vitro* toxicology models. Furthermore, this advancement aligns with the 3R principles in animal testing, reinforcing the potential of *in vitro* alternatives as standardized replacements for *in vivo* ocular irritation assays.

This study provides a focused structural and viability assessment of bovine corneas preserved under accessible low-temperature conditions. While the BCOP assay itself was not performed, the preservation strategy was designed to meet its structural and physiological requirements. The next critical step will be functional validation through direct comparison of opacity and permeability responses to benchmark chemicals, as outlined in OECD Test Guideline 437. In this context, our results support future studies aimed at confirming the regulatory applicability of this preservation strategy in *in vitro* ocular irritation models.

## Conclusions

This study demonstrates that bovine corneas can be structurally preserved for up to 30 days using 10% DMSO at −20 °C. Among the tested conditions, this approach provided the most consistent maintenance of corneal transparency, histological architecture, and retention of a viable endothelial cell population. The use of a conventional laboratory freezer, rather than ultralow-temperature equipment, represents a cost-effective and accessible option that may extend the usability of excised corneas, particularly in laboratories with limited infrastructure. While these findings represent an important step toward supporting *in vitro* ocular toxicology models and advancing the 3R principles, the preservation strategy described here should be regarded as a foundational approach. Further functional validation with reference chemicals will be essential before this method can be considered for routine regulatory applications.

## Supplementary Material



## Notes

Figures (Figures S1–S6) and tables (Tables S1–S5) that provide detailed statistical modeling data, complete quantitative histological scores, and additional representative macroscopic and microscopic images to support the analyses presented in the main text (PDF)

## Abbreviations

3R’s: reduction, refinement, and replacement
AICc: Akaike Information Criterion corrected for small samples
BS: bovine serum
BSA: bovine serum albumin
CPA: cryoprotective agent
DMEM: Dulbecco’s modified Eagle medium
DMSO: dimethyl sulfoxide
EG: ethylene glycol
HE: hematoxylin and eosin
I-PVP: iodopolyvidone
LM: linear model
NaCl: sodium chloride
OECD: Organisation for Economic Cooperation and Development
PBS: phosphate-buffered saline
R: statistical software R
SEM: standard error of the mean
