# Supplementary material for: Structural and Viability Assessment of Bovine Corneas Preserved at an Accessible Low-Temperature (−20 °C) for Eye Irritation Models
Source: ACS Omega. 2025 Oct 3;10(40):46759–68. doi: 10.1021/acsomega.5c04314 (PMC12529383; doi:10.1021/acsomega.5c04314)
Supplement: Supplementary file 1 [file ao5c04314_si_001.pdf]

Supporting information

Structural and Viability Assessment of Bovine Corneas Preserved at an Accessible Low-Temperature (–20 °C) for Eye Irritation Models

Geovana Onorato[a], Janildo Ludolf Reis Junior[b], Humberto Mello Brandão[c] and Michele Munka,[a]*

Laboratory of Nanobiotechnology and Nanotoxicology, Department of Biology, Federal University of Juiz de Fora, Juiz de Fora, 36036-900, Minas Gerais, Brazil.

[b] Department of Veterinary Medicine, Federal University of Juiz de Fora, Juiz de Fora, 36036-900, Minas Gerais, Brazil.

[c] Laboratory of Applied Nanotechnology for Animal Production and Health, Brazilian Agricultural Research Corporation, 36038-330, Juiz de Fora, Minas Gerais, Brazil.

[*] Corresponding author: Email: michele.munk@ufjf.br

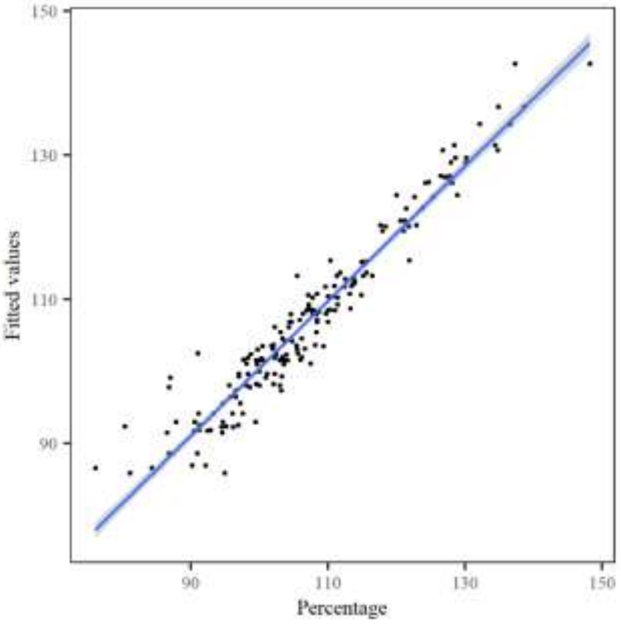

Figure S1. Linear regression analysis of corneal weight variation after exposure to cryoprotective agents (CPAs). The x-axis shows the observed relative weight (%) and the y-axis displays the corresponding fitted values. The blue line represents the regression line, and the shaded area denotes the 95% confidence interval. The strong linear relationship indicates consistent weight variation patterns across CPA treatments.

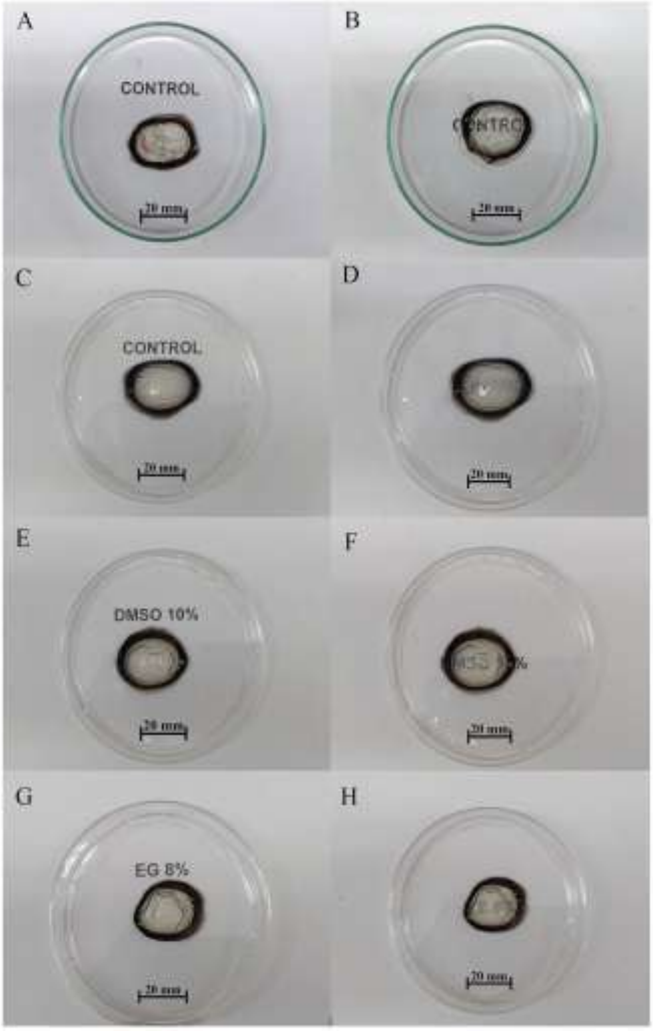

Figure S2. Representative macroscopic images of fresh corneas (A, B) and corneas preserved at -20 °C under different conditions. Panels C and D show frozen controls without cryoprotectants; E and F show corneas treated with 10% (v/v) DMSO; and G and H show corneas treated with 8% (v/v) EG. Increased opacity and surface irregularities were observed in frozen groups compared to fresh controls. Scale bar: 20 mm.

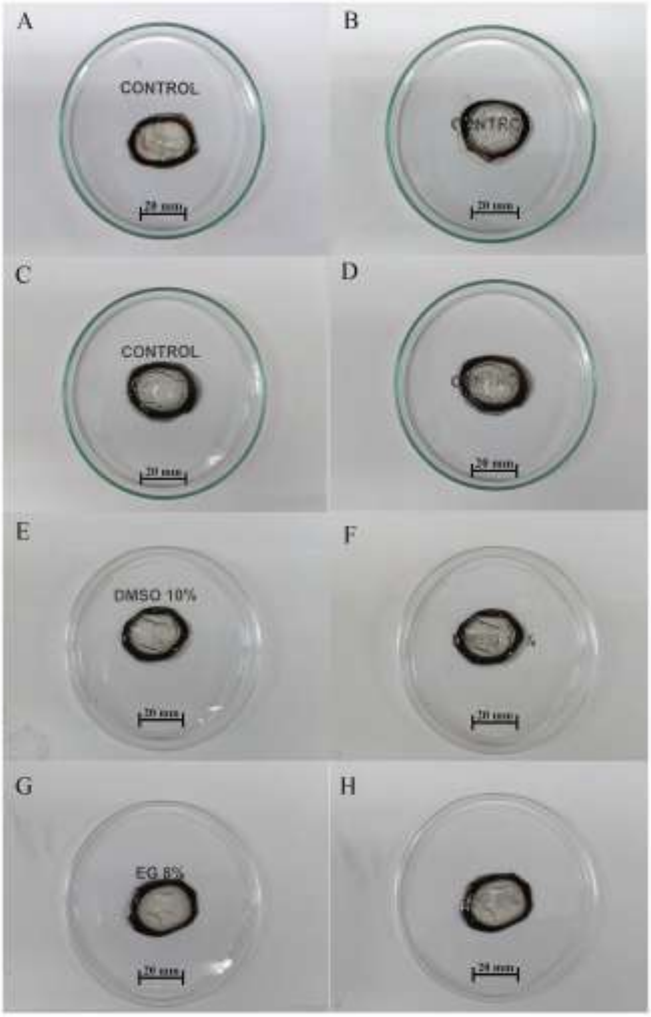

Figure S3. Representative macroscopic images of fresh corneas (A, B) and corneas preserved at -80 °C under different conditions. Panels C and D show frozen controls without cryoprotectants; E and F show corneas treated with 10% (v/v) DMSO; and G and H show corneas treated with 8% (v/v) EG. Compared to fresh tissues, frozen corneas exhibit increased opacity and decreased surface uniformity, particularly in the EG-treated group. Scale bar: 20 mm.

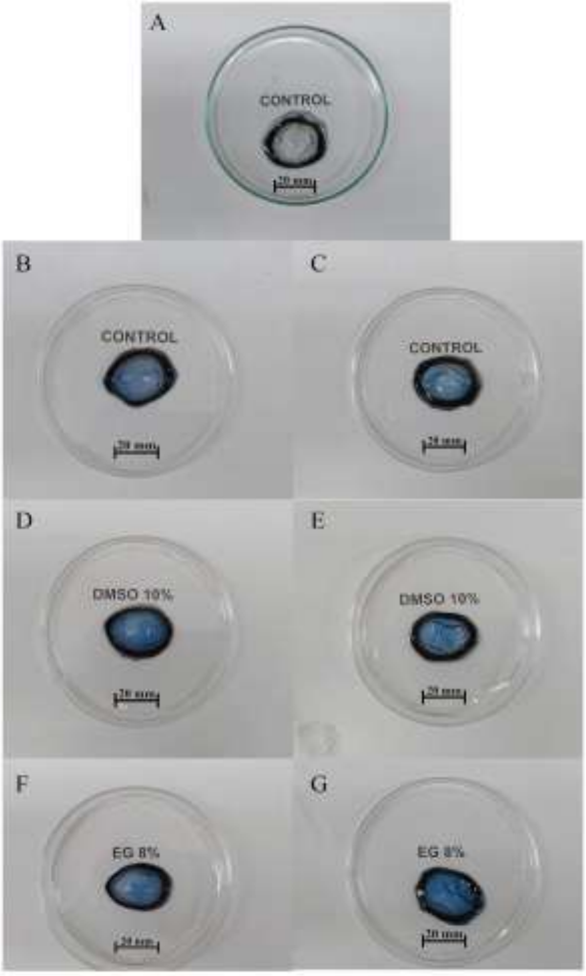

Figure S4. Representative images of Trypan Blue staining in fresh corneas (A) and corneas cryopreserved at -20 °C (B, D, F) or −80 °C (C, E, G). Panels B and C show frozen controls without cryoprotectants; D and E correspond to corneas treated with 10% (v/v) DMSO; and F and G show those treated with 8% (v/v) EG. Trypan Blue staining indicates compromised membrane integrity, with increased uptake reflecting cell damage. Scale bar: 20 mm.

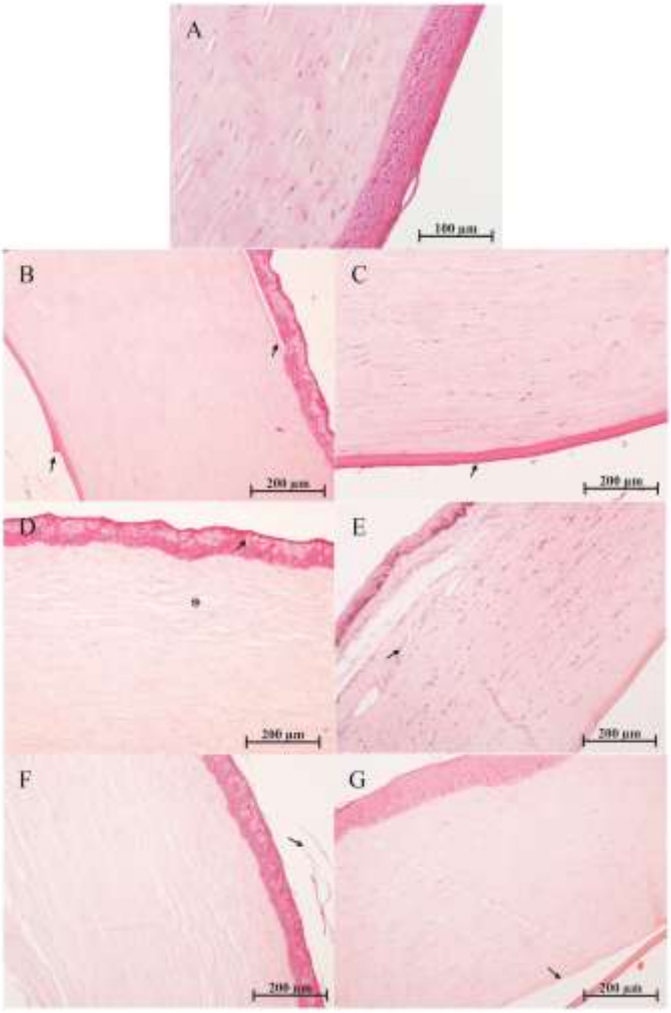

Figure S5. Representative histological sections of fresh corneas (A) and control-treated corneas after cryopreservation at -20 °C (B, D, F) or -80 °C (C, E, G). At -20 °C, typical alterations included epithelial detachment (B), stromal clefts (D, asterisks), and epithelial vacuolization (F). At -80 °C, additional structural damage was observed, such as endothelial cell detachment (C), persistent stromal clefts (E), and Descemet's membrane separation (G, asterisks). Arrows highlight affected regions. Hematoxylin and eosin (H&E) staining. Scale bars: 100 or 200 µm.

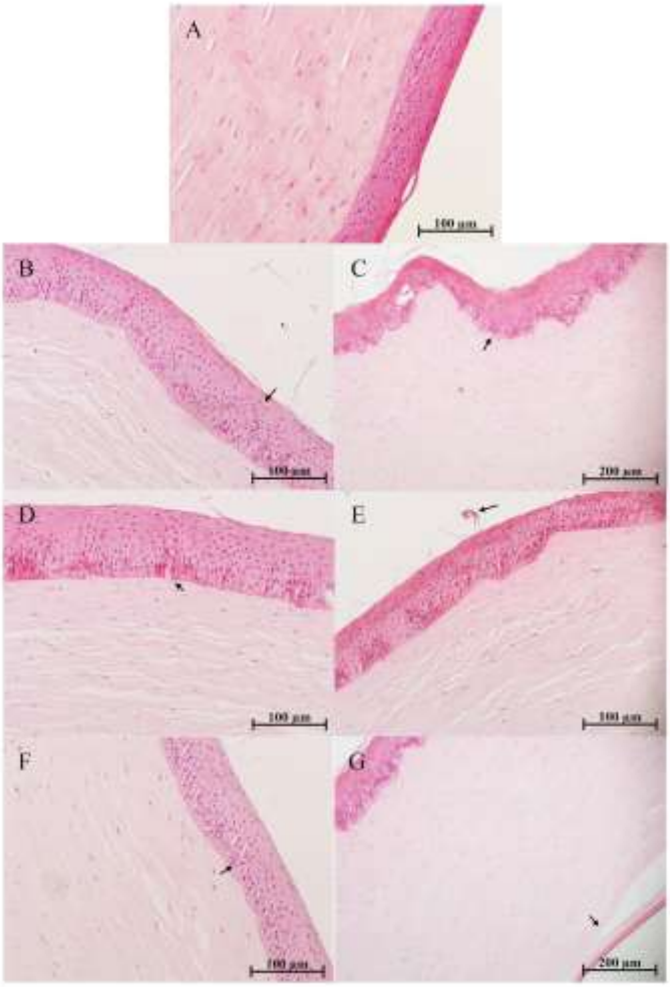

Figure S6. Representative histological sections of fresh corneas (A) and EG-treated corneas after low-temperature freezing at -20 °C (B, D, F) and -80 °C (C, E, G). At -20 °C, common alterations included epithelial detachment (B), stromal cleft formation (B, D), and epithelial vacuolization (F). At -80 °C, more severe changes were observed, including pronounced epithelial disorganization (C), extensive Descemet's membrane detachment (E), and severe endothelial disorganization (G). Arrows indicate regions of morphological disruption. Hematoxylin and eosin (H&E) staining. Scale bars: 100 or 200 µm.

Table S1. Alternative Linear Models with R^2 and Akaike Information Criterion (AICc) Values*

Linear models	R^2	AIC
Por = Trat + Conc_t + Adj+ T + Conc_t:Adj + Trat:T + Adj:T	0.91	1260.22
Por = Trat*Conc_t * Adj * T	0.90	1372.32
Por = Trat + Adj + T	0.74	1415.43
Por = Trat+Conc_t+ Adj + T	0.73	1417.37

* Four linear models were tested to identify the best-fitting statistical approach for analyzing percentage weight variation (Por) in bovine corneas following exposure to different cryoprotectants (CPAs) and adjuvants over time. Models included combinations of treatment (Trat), concentration (Conc_t), adjuvant (Adj), time (T), and interaction terms. The coefficient of determination (R^2) indicates the proportion of variance explained by each model, and the Akaike Information Criterion corrected for small samples (AICc) was used to compare model parsimony. The model with the lowest AICc and highest R^2 (first row) was selected as the most robust for further analyses.

Table S2. Percentage distribution of histological scores for superficial corneal epithelium alterations and pseudoparakeratinization after freezing.*

Parameter	Treatment	Freezing Temperature	Absent (%)	Minimal (%)	Discreet (%)	Discreet to Moderate (%)	Moderate (%)	Moderate to Accentuated (%)	Accentuated (%)
**Superficial Lamellar Detachment of Epithelium**	Control	-20°C	22	44	33	0	0	0	0
	DMSO		11	67	22	0	0	0	0
	EG		0	0	78	0	0	0	0
	Control	-80°C	44	33	22	0	0	0	0
	DMSO		25	25	50	0	0	0	0
	EG		0	0	78	0	22	0	0
**Superficial Keratinocytand Vacuolization**	Control	-20°C	11	0	33	0	22	0	33
	DMSO		0	0	44	44	11	0	0
	EG		0	0	44	11	44	0	0
	Control	-80C	67	11	22	0	0	0	0
	DMSO		0	38	25	25	13	0	0
	EG		0	0	67	0	11	0	22
**Pseudo Parakeratinization**	Control	-20°C	0	100	0	0	0	0	0
	DMSO		0	11	89	0	0	0	0
	EG		56	0	44	0	0	0	0
	Control	-80°C	67	33	0	0	0	0	0
	DMSO		75	13	13	0	0	0	0
	EG		100	0	0	0	0	0	0

* Histological assessment was conducted for three epithelial parameters: (i) superficial lamellar detachment of the epithelium, (ii) vacuolization of superficial keratinocytes, and (iii) pseudo-parakeratinization. Corneal samples were stained with hematoxylin and eosin and scored using a seven-level semi-quantitative scale: Absent, Minimal, Discreet, Discreet to Moderate, Moderate, Moderate to Accentuated, and Accentuated. Data are expressed as the percentage (%) of samples falling within each category for each treatment condition (Control, DMSO, EG) and freezing temperature (-20°C and -80°C), with n = 9 per group. This analysis provides a comparative overview of superficial epithelial damage under different cryopreservation conditions.

Tabel S3. Percentage distribution of histological scores for alterations in the basal layer of the corneal epithelium after freezing *

Parameter	Treatment	Freezing Temperature	Absent (%)	Minimal (%)	Discreet (%)	Discreet to Moderate (%)	Moderate (%)	Moderate to Accentuated (%)	Accentuated (%)
**Focal Detachment of Epithelium/Stroma**	Control	-20°C	44	0	22	22	11	0	0
	DMSO		89	0	0	0	11	0	0
	EG		33	0	22	11	0	0	33
	Control	-80°C	67	0	11	11	11	0	0
	DMSO		38	0	0	13	38	0	13
	EG		44	0	11	0	44	0	0
**Cytoplasmic and Nuclear Retraction/Deformity of Basal Keratinocytes**	Control	-20°C	0	0	0	0	67	0	33
	DMSO		22	33	22	22	0	0	0
	EG		0	33	11	22	33	0	0
	Control	-80°C	0	67	11	22	0	0	0
	DMSO		0	25	13	13	38	0	13
	EG		0	0	78	0	22	0	0
**Vacuolization of Basal Keratinocytes**	Control	-20°C	56	0	0	0	22	0	22
	DMSO		78	0	0	11	11	0	0
	EG		0	0	11	11	22	33	22
	Control	-80°C	89	0	0	11	0	0	0
	DMSO		0	0	38	25	38	0	0
	EG		0	0	33	0	22	22	22

* Histological sections stained with hematoxylin and eosin were evaluated for three epithelial parameters: (i) focal detachment of the epithelium from the stroma, (ii) cytoplasmic and nuclear retraction or deformity in basal keratinocytes, and (iii) vacuolization of basal keratinocytes. Each parameter was classified into seven levels of severity: Absent, Minimal, Discreet, Discreet to Moderate, Moderate, Moderate to Accentuated, and Accentuated. Data are presented as the percentage (%) of samples in each category for each treatment group (Control, DMSO, EG) at two freezing temperatures (-20°C and -80°C), with n = 9 per group. This analysis provides a comparative overview of epithelial integrity under different cryopreservation conditions.

Table S4. Formation of unstained clefts dissociating the stroma*

Treatment	Freezing Temperature	Absent (%)	Minimal (%)	Discreet (%)	Discreet to Moderate (%)	Moderate (%)	Moderate to Accentuated (%)	Accentuated (%)
**Control**	-20°C	-	-	-	56	33	-	11
**DMSO**		-	-	33	11	33	-	22
**EG**		-	-	11	-	56	33	-
**Control**	-80°C	-	-	44	33	22	-	-
**DMSO**		13	25	13	25	13	-	13
**EG**		-	-	33	22	11	33	-

* The presence and severity of unstained clefts disrupting stromal integrity were assessed in hematoxylin and eosin-stained histological sections. Samples were classified into seven damage categories: Absent, Minimal, Discreet, Discreet to Moderate, Moderate, Moderate to Accentuated, and Accentuated. Results are expressed as the percentage (%) of corneas in each category for each treatment group (Control, DMSO, EG) stored at -20°C or -80°C (n = 9 per group). This analysis reflects the degree of stromal dissociation induced by different cryoprotective agents and storage conditions.

Table S5. Semi-quantitative classification of histological damage in cryopreserved bovine corneas under different treatments and freezing temperatures.*

Treatment	Freezing Temperature	Absent (%)	Minimal (%)	Discreet (%)	Discreet to Moderate (%)	Moderate (%)	Moderate to Accentuated (%)	Accentuated (%)
**Control**	-20°C	-	-	-	11	56	-	33
**DMSO**		-	44	44	-	11	-	-
**EG**		-	11	-	-	22	56	11
**Control**	-80°C	-	-	33	11	56	-	-
**DMSO**		-	25	50	-	25	-	-
**EG**		22	-	11	-	44	-	22

* Corneal samples were evaluated based on a seven-level semi-quantitative scale: Absent, Minimal, Discreet, Discreet to Moderate, Moderate, Moderate to Accentuated, and Accentuated. This classification reflects the degree of structural alterations in the epithelial, stromal, and endothelial layers observed in hematoxylin and eosin-stained sections. Results are expressed as the percentage (%) of corneas in each damage category for each treatment group (Control, DMSO, EG) stored at either -20°C or -80°C (n = 9 per group).
